# Supplementary material for: Exposure to mold proteases stimulates mucin production in airway epithelial cells through Ras/Raf1/ERK signal pathway
Source: PLoS One. 2020 Apr 22;15(4):e0231990. doi: 10.1371/journal.pone.0231990 (PMC7176129; doi:10.1371/journal.pone.0231990)
Supplement: S1 Fig — (PDF) [file pone.0231990.s001.pdf]

Supplementary Materials

Cell viability assay

NCI-H292 cell viability was analyzed by using CellTiter 96 Non-Radioactive Cell Proliferation Assay Kit (Promega, Madison, WI). According to the manufacturer's instruction, NCI-H292 cells were seeded into a 96 well cell culture microplate at a density of $4x10^4$ cells/well. After serum starving, the cells were treated with different doses of AG1478, Trypsin and DMSO for 6 hrs and then 120 µl of MTT buffer was added per well. The absorbance was measured at 490 nm using a plate reader (Tecan, Männedorf, Switzerland). The percentage of viability was calculated as: (treatment/control) *100. The control was designated as 100% viability.

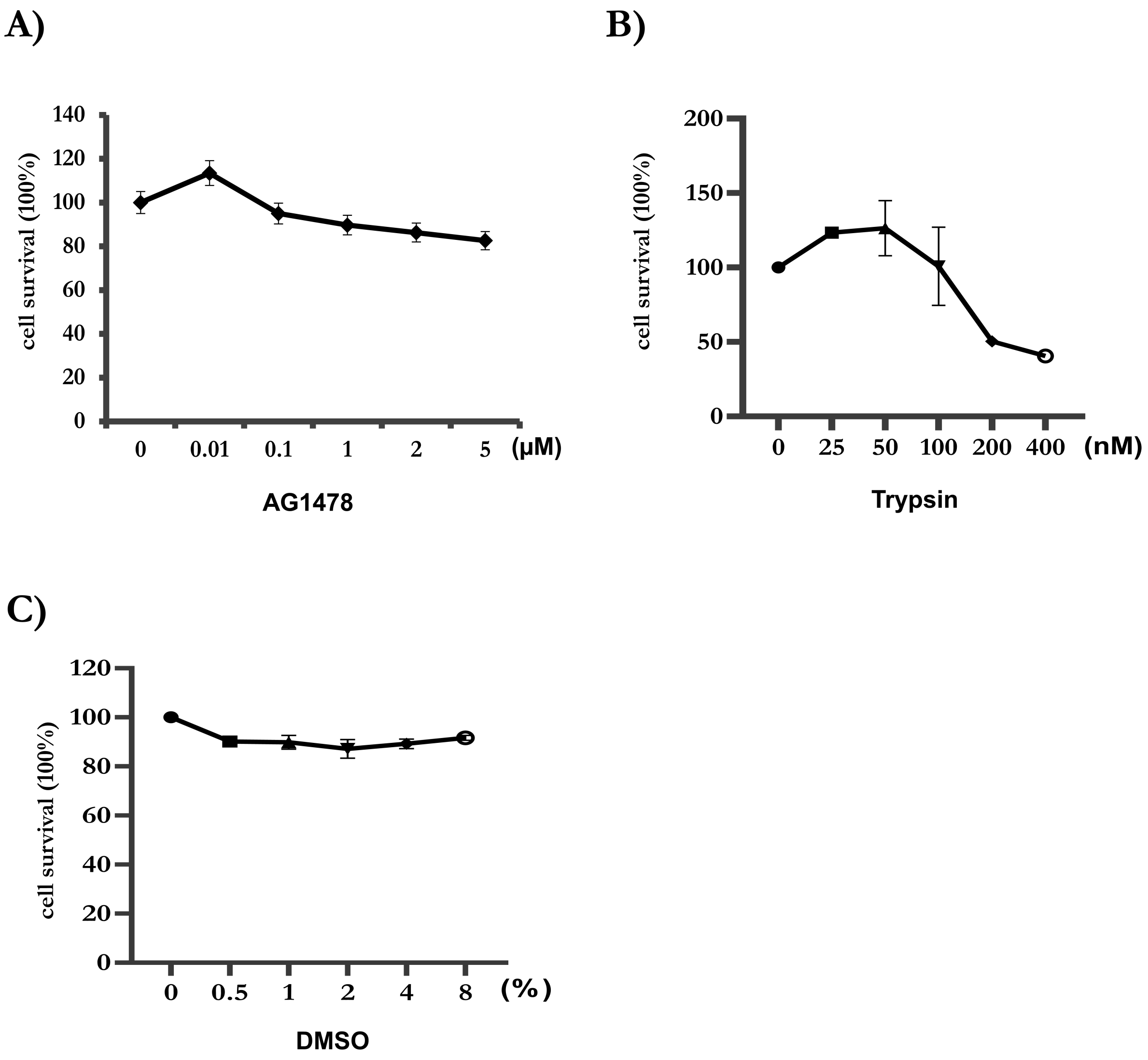

S1 Fig. (A) NCI-H292 cells were stimulated with 0.01 μM, 0.1 μM, 1 μM, 2 μM, 5 μM AG1478 for 6 hrs. (B) The cells were stimulated with 25 nM, 50 nM, 100 nM, 200 nM, 400 nM Trypsin for 6 hrs. (C) The cells were stimulated with 0.5%, 1%, 2%, 4%, 8% DMSO for 6 hrs.
