# Supplementary material for: Exposure to mold proteases stimulates mucin production in airway epithelial cells through Ras/Raf1/ERK signal pathway
Source: PLoS One. 2020 Apr 22;15(4):e0231990. doi: 10.1371/journal.pone.0231990 (PMC7176129; doi:10.1371/journal.pone.0231990)
Supplement: S2 Fig — (PDF) [file pone.0231990.s002.pdf]

Supplementary Materials

Measurement of cellular ROS

NCI-H292 cells were plated into 96-well plates and grown overnight. Cells were washed with OPTI-MEM for two times and incubated with 3 μM of CM-H2DCFDA (Invitrogen, Carlsbad, CA) for 30 min. Cells were then treated with 7.5 μg/ml AFE in OPTI-MEM. The fluorescence was detected every 5 min by a plate reader (Tecan Infinite® M1000, Piedmont, NC). Background reading from the cells that were not loaded with CM-H2DCFDA was used as a blank.

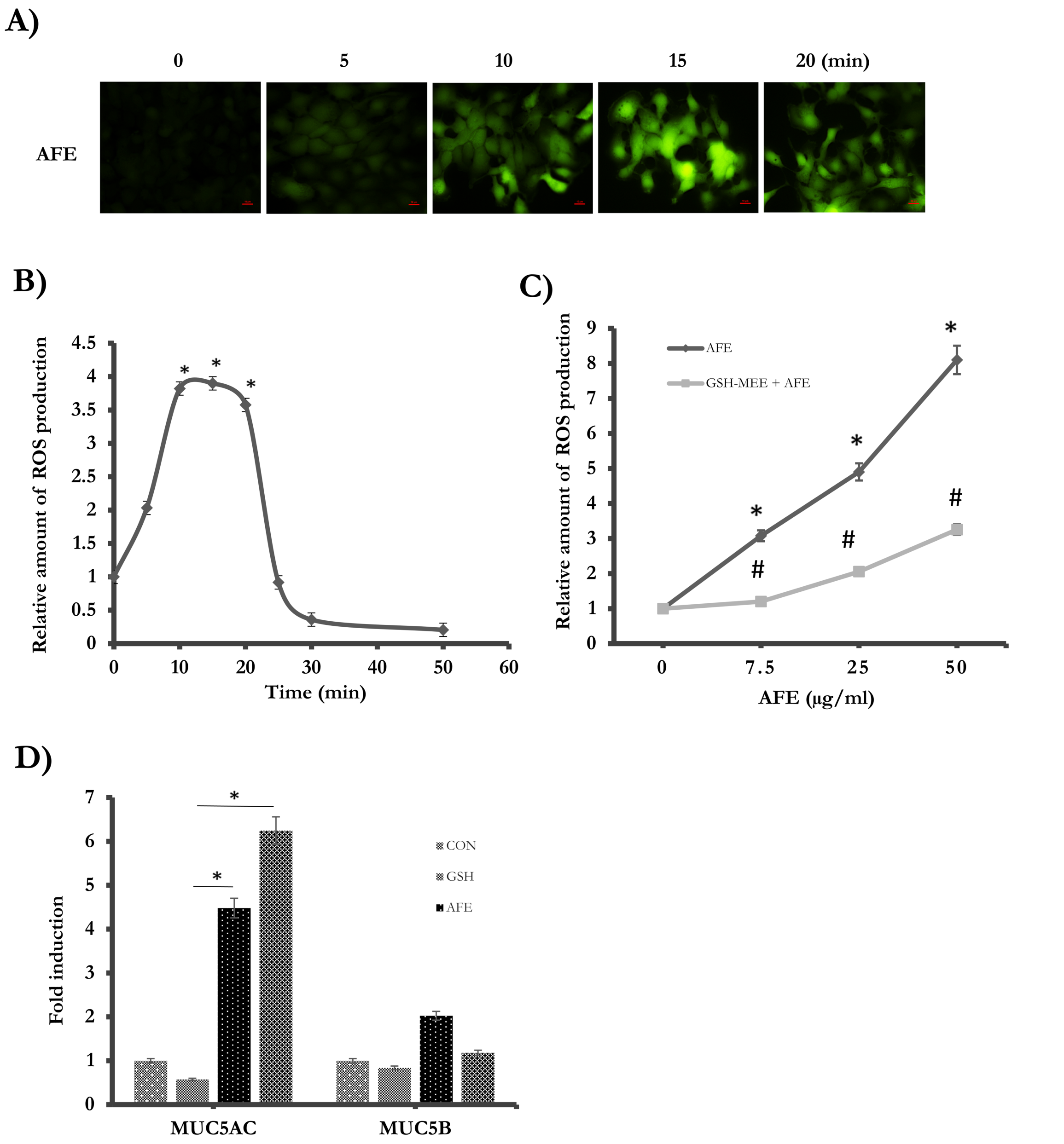

S2 Fig. (A) NCI-H292 cells were stimulated with 7.5 μg/ml AFE and intracellular ROS generation was measured every 5 min. (B) Quantification of ROS generation. n=5. (C) Application of GSH-MEE significantly blocked the dose-dependent AFE-induced ROS generation. (D) The cells were pre-treated with 5 mM GSH for 1 hr, and then treated with AFE for 6 hrs. MUC5AC and MUC5B were quantified by Real-Time PCR.*: AFE vs control (0 ug/ml) $P < 0.05$. [#:] AFE vs GSH-MEE +AFE, $P < 0.05$. n=4.
