# Supplementary material for: Exposure to mold proteases stimulates mucin production in airway epithelial cells through Ras/Raf1/ERK signal pathway
Source: PLoS One. 2020 Apr 22;15(4):e0231990. doi: 10.1371/journal.pone.0231990 (PMC7176129; doi:10.1371/journal.pone.0231990)
Supplement: S1 Raw Images — (PDF) [file pone.0231990.s003.pdf]

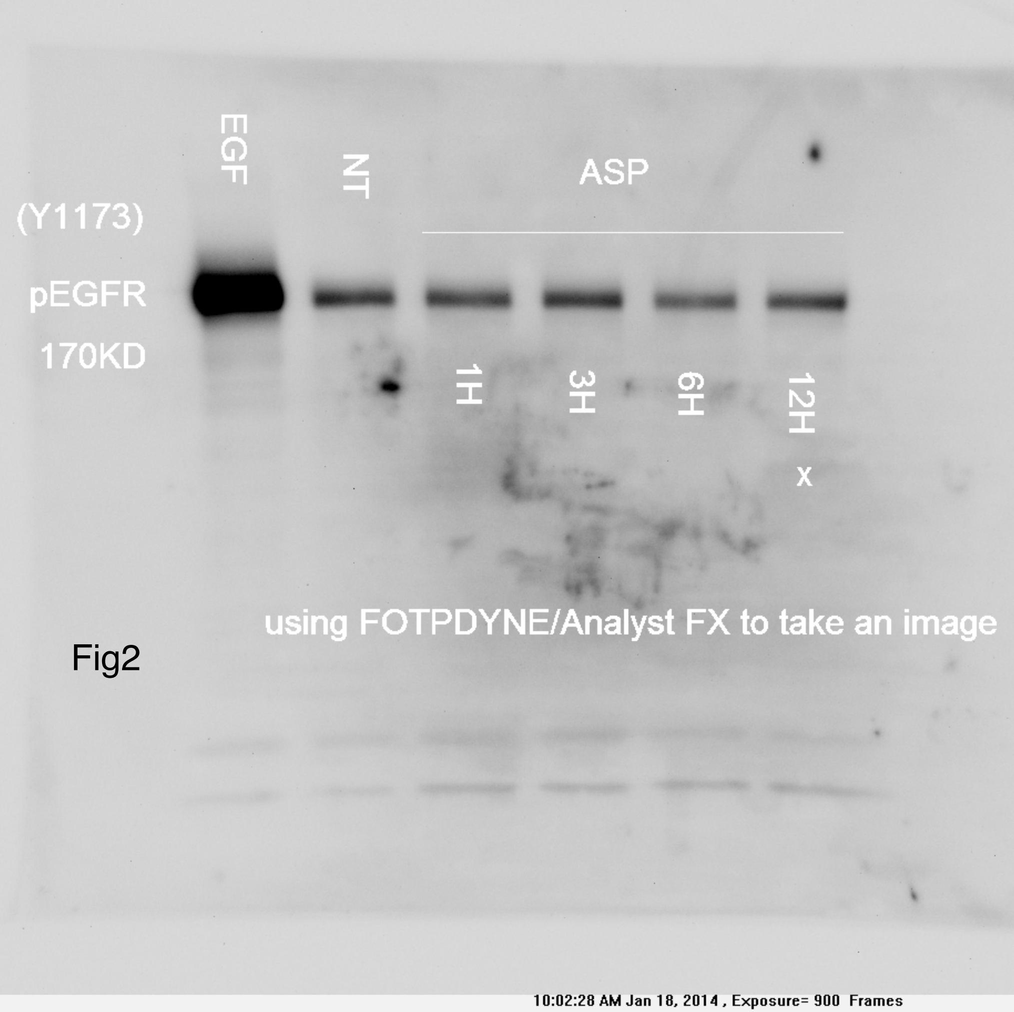
EGF
NT
ASP
(Y1173)
pEGFR
170KD
1H
3H
6H
12H
x
using FOTPDYNE/Analyst FX to take an image
10:02:28 AM Jan 18, 2014 , Exposure= 900 Frames

Fig2

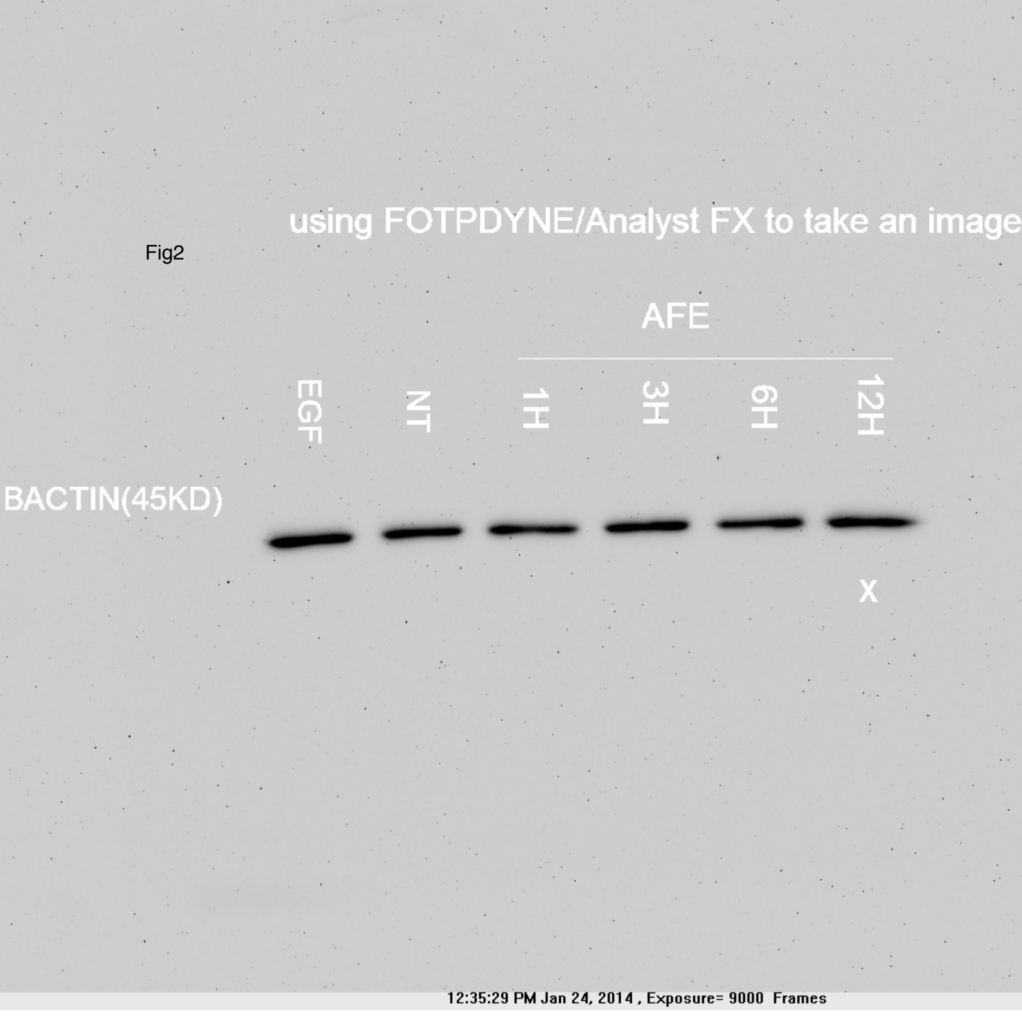
using FOTPDYNE/Analyst FX to take an image
AFE
EGF
NT
1H
3H
6H
12H
BACTIN(45KD)
X
12:35:29 PM Jan 24, 2014 , Exposure= 9000 Frames

Fig2

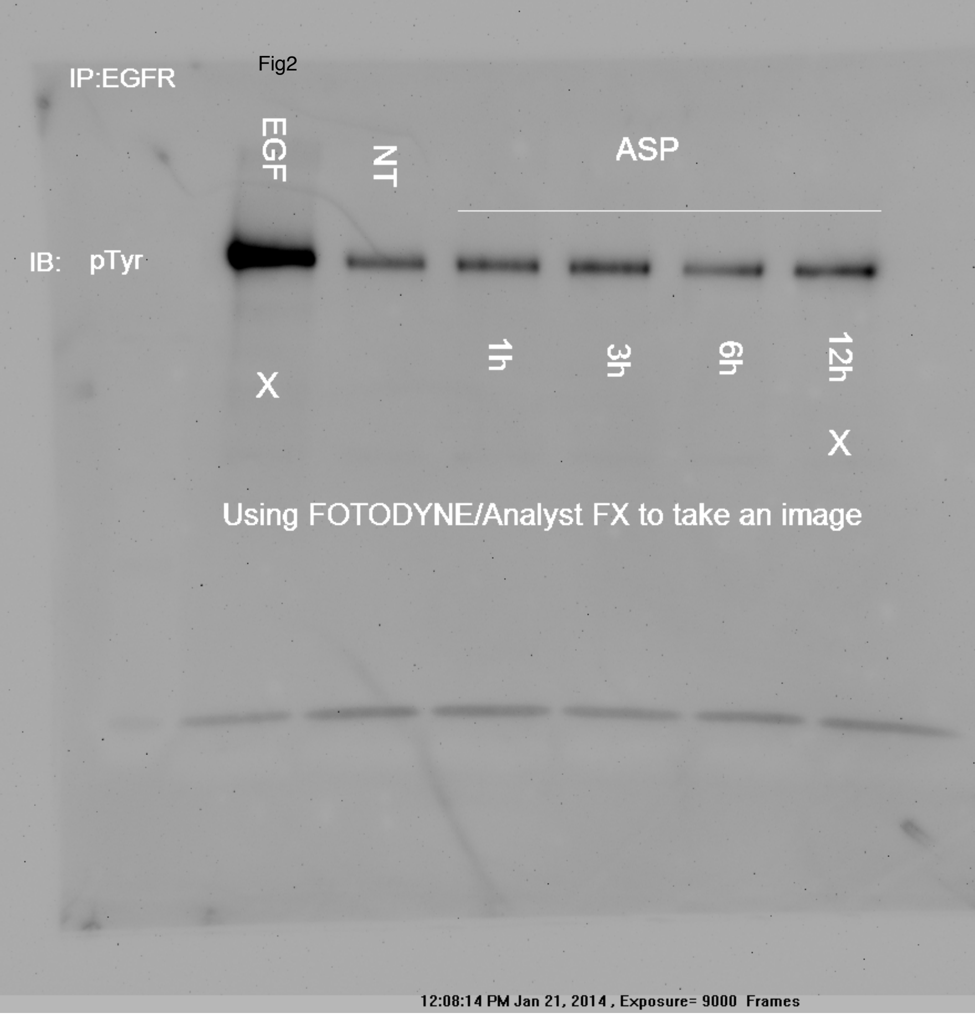

Fig2
IP:EGFR
EGF
NT
ASP
IB: pTyr
1h
3h
6h
12h
X
X
Using FOTODYNE/Analyst FX to take an image
12:08:14 PM Jan 21, 2014 , Exposure= 9000 Frames

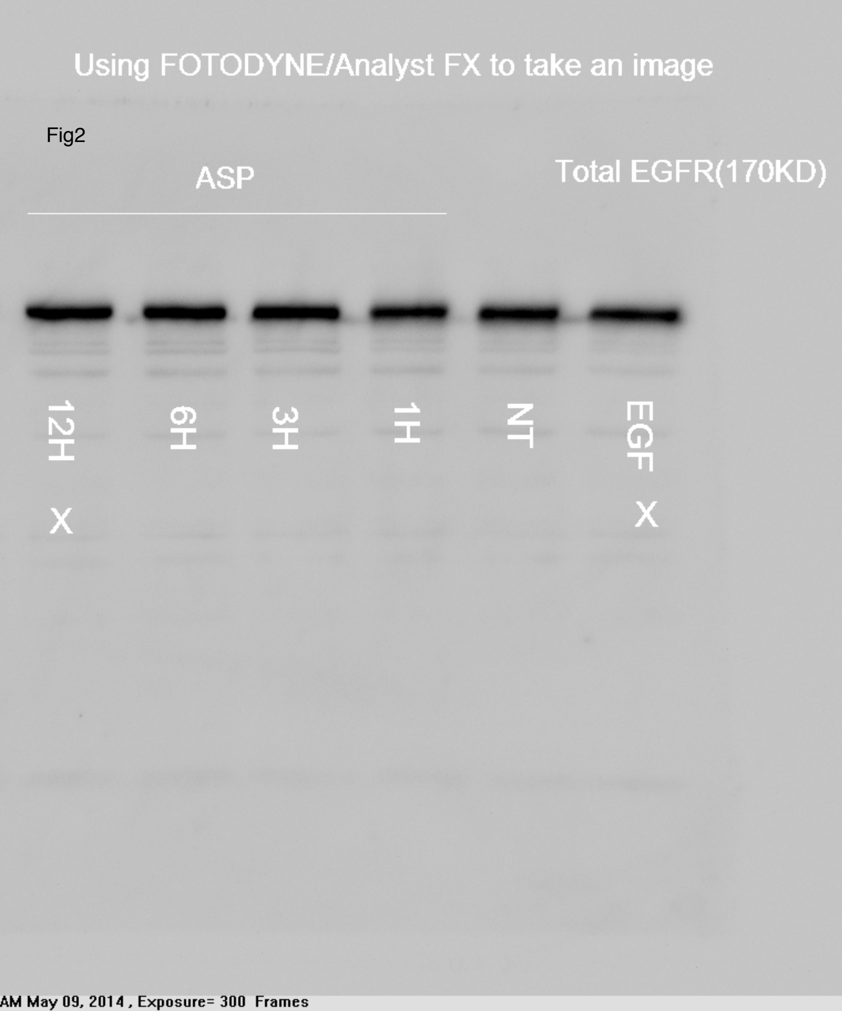
Using FOTODYNE/Analyst FX to take an image
Fig2
ASP
Total EGFR(170KD)
12H
6H
3H
1H
NT
EGF
X
X
AM May 09, 2014 , Exposure= 300 Frames

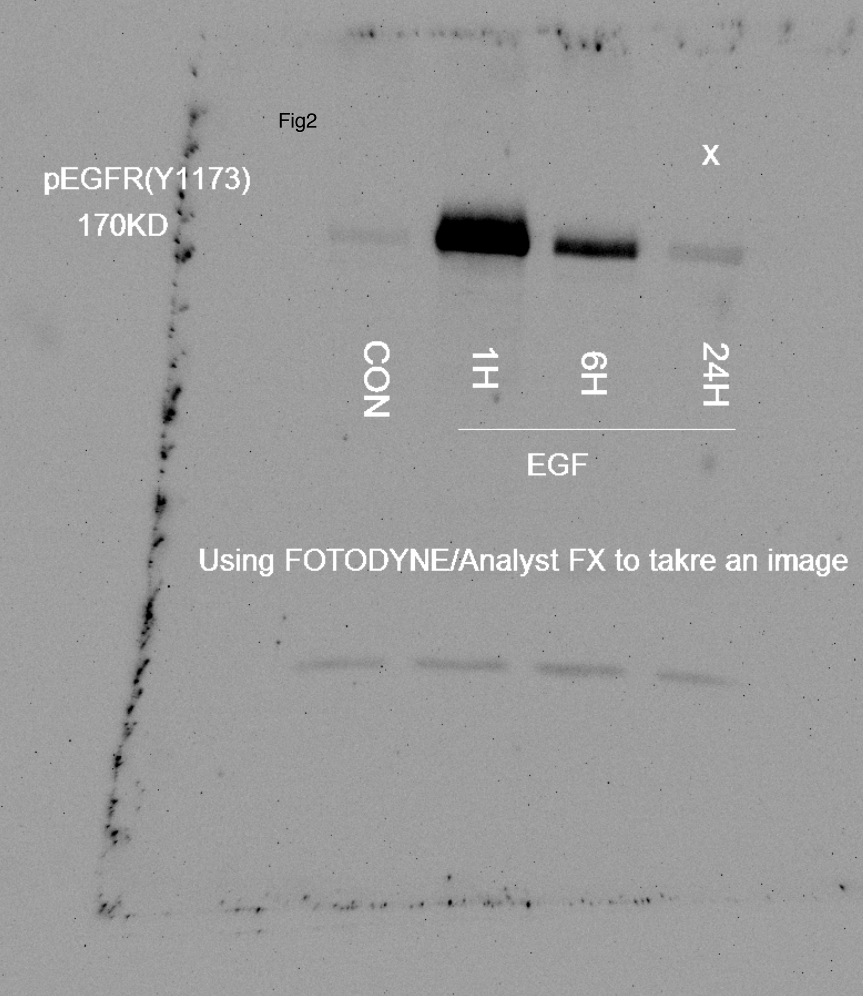
Fig2
pEGFR(Y1173)
170KD
X
CON
1H
6H
24H
EGF
Using FOTODYNE/Analyst FX to takre an image

Fig2

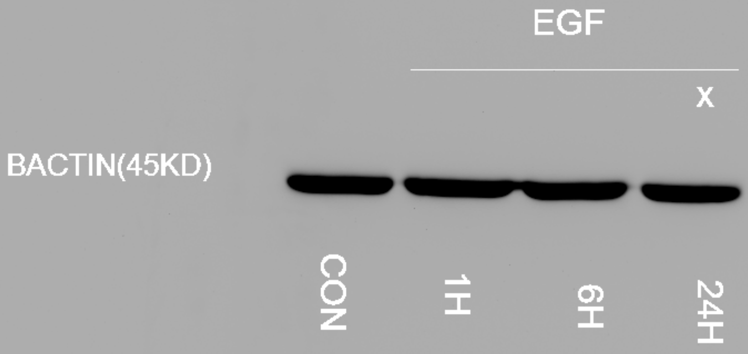

Using FOTODYNE/Analyst FX to takre an image

12:08:35 PM Sep 27, 2019 , Exposure= 1800 Frames

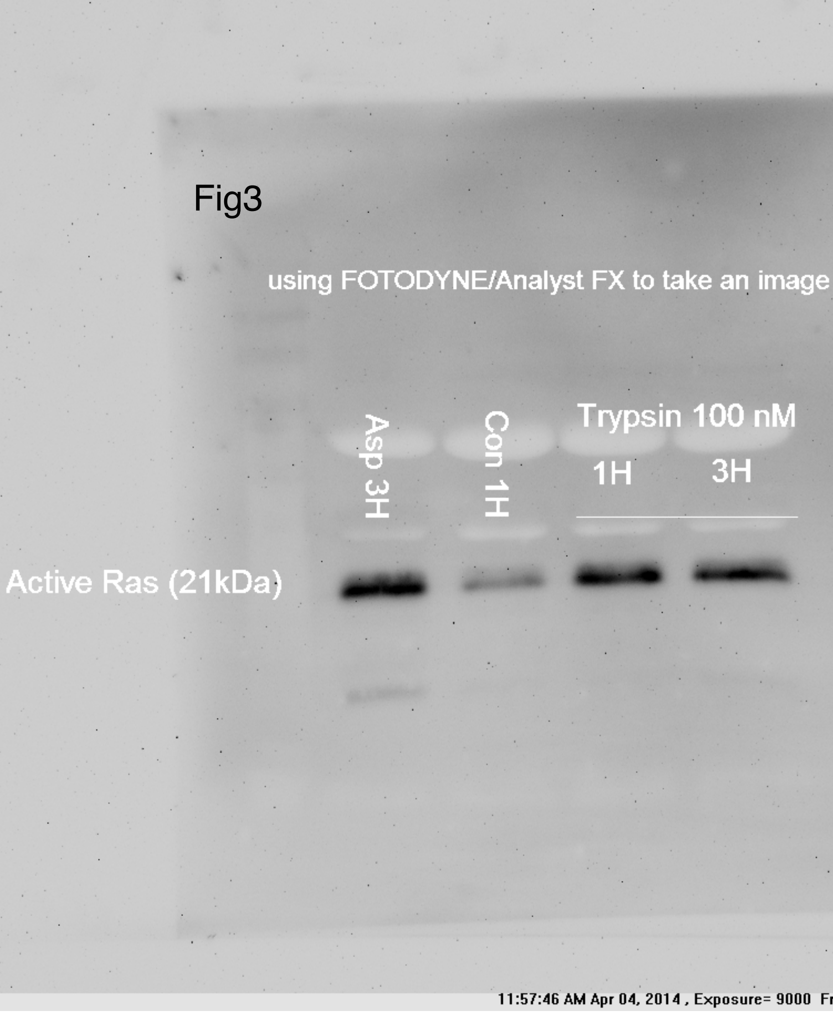
Fig3
using FOTODYNE/Analyst FX to take an image
Asp 3H
Con 1H
Trypsin 100 nM
1H
3H
Active Ras (21kDa)
11:57:46 AM Apr 04, 2014 , Exposure= 9000 Fr

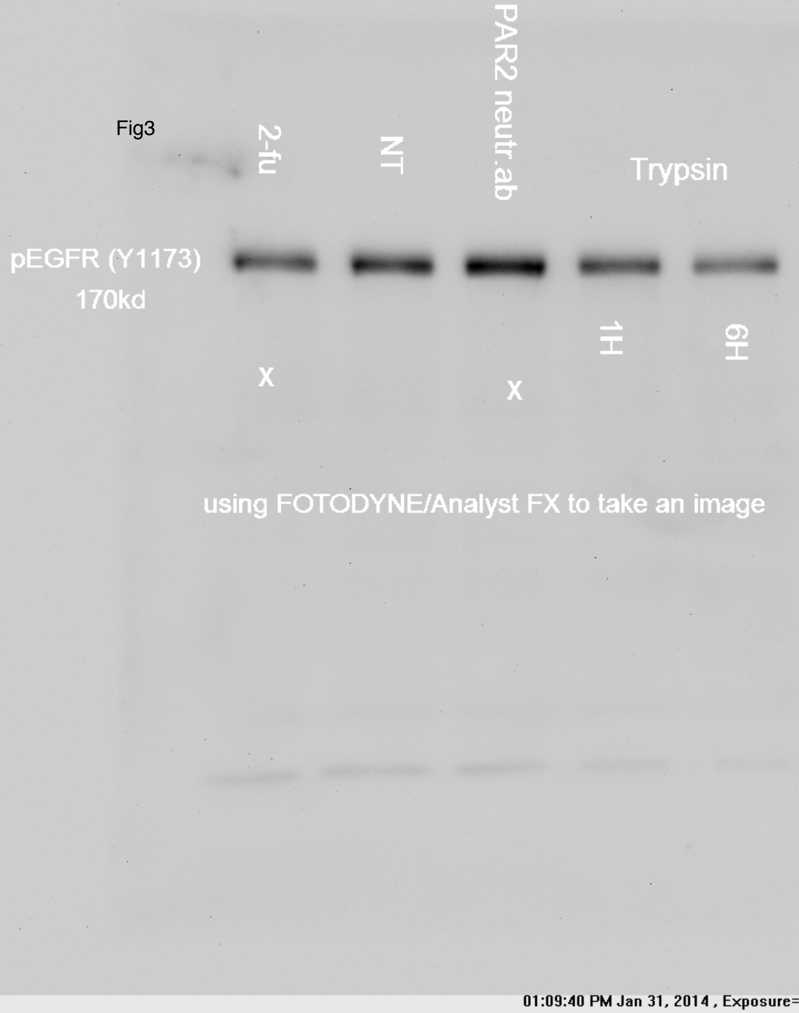
Fig3
2-fu
NT
PAR2 neutr.ab
Trypsin
pEGFR (Y1173)
170kd
1H
6H
X
X
using FOTODYNE/Analyst FX to take an image
01:09:40 PM Jan 31, 2014 , Exposure=

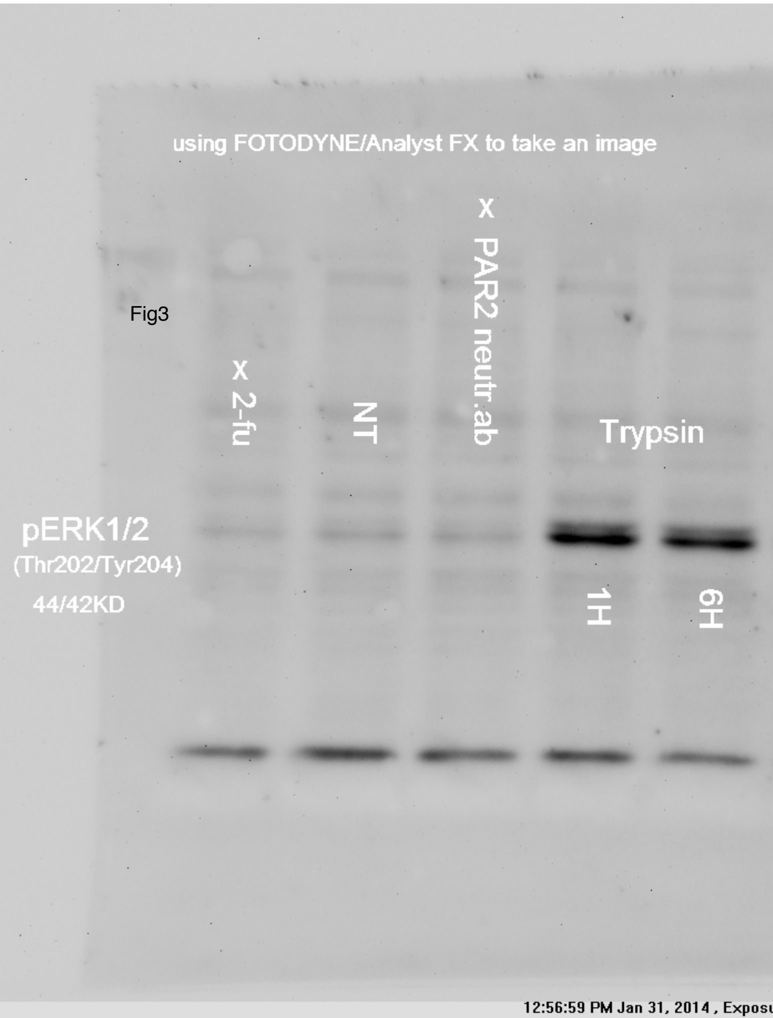
using FOTODYNE/Analyst FX to take an image
Fig3
x 2-fu
NT
x PAR2 neutr.ab
Trypsin
pERK1/2
(Thr202/Tyr204)
44/42KD
1H
6H
12:56:59 PM Jan 31, 2014 , Exposu

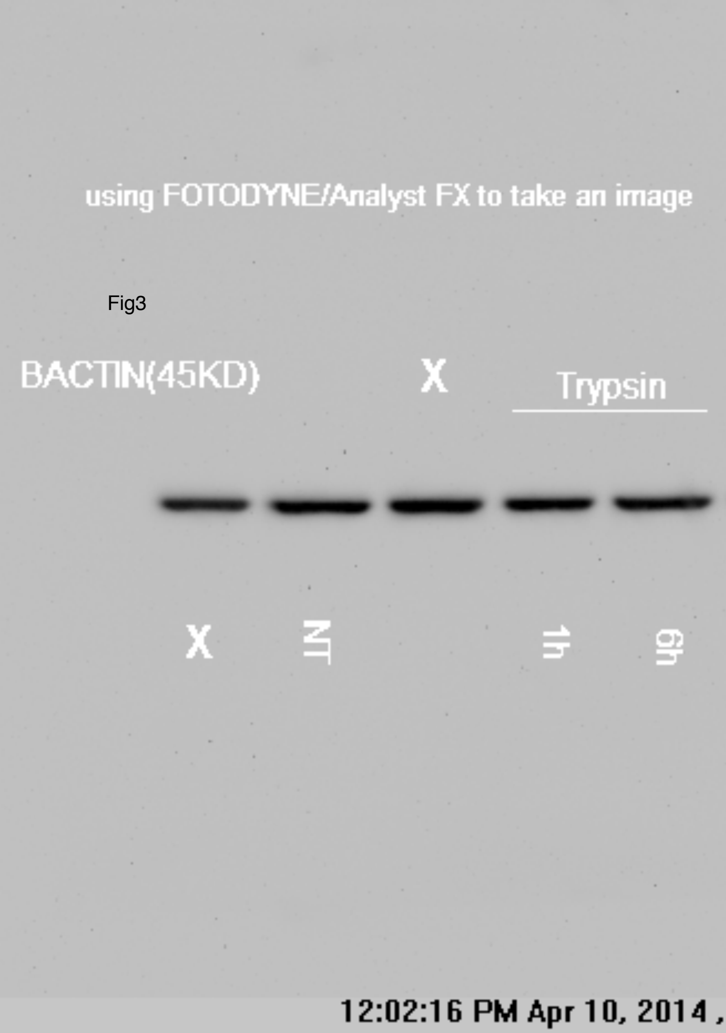
using FOTODYNE/Analyst FX to take an image
BACTIN(45KD)
X
Trypsin
X
NT
1h
6h
12:02:16 PM Apr 10, 2014 ,
Fig3

Fig4

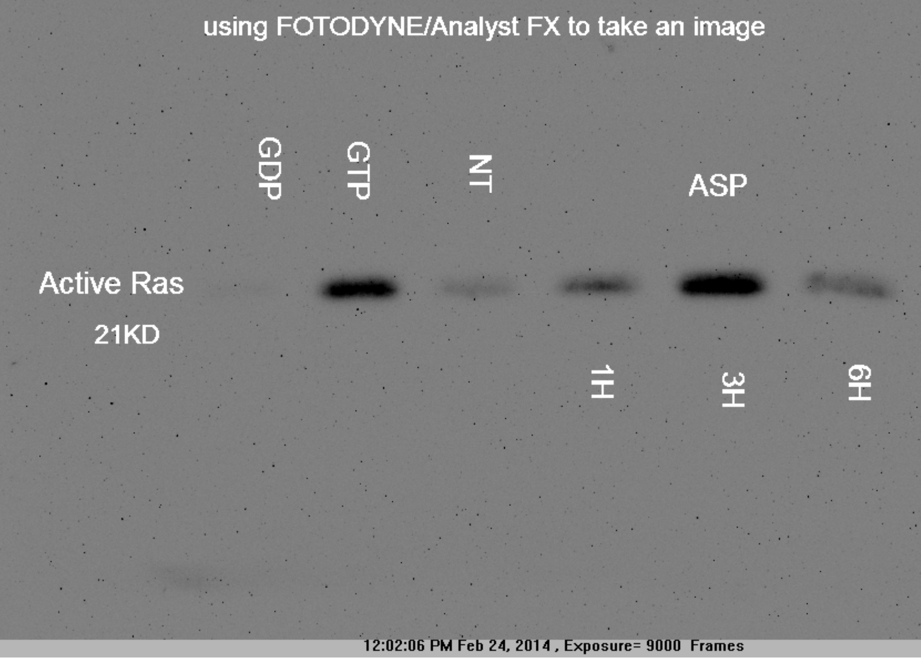
using FOTODYNE/Analyst FX to take an image
GDP
GTP
NT
ASP
Active Ras
21KD
1H
3H
6H
12:02:06 PM Feb 24, 2014 , Exposure= 9000 Frames

Fig4

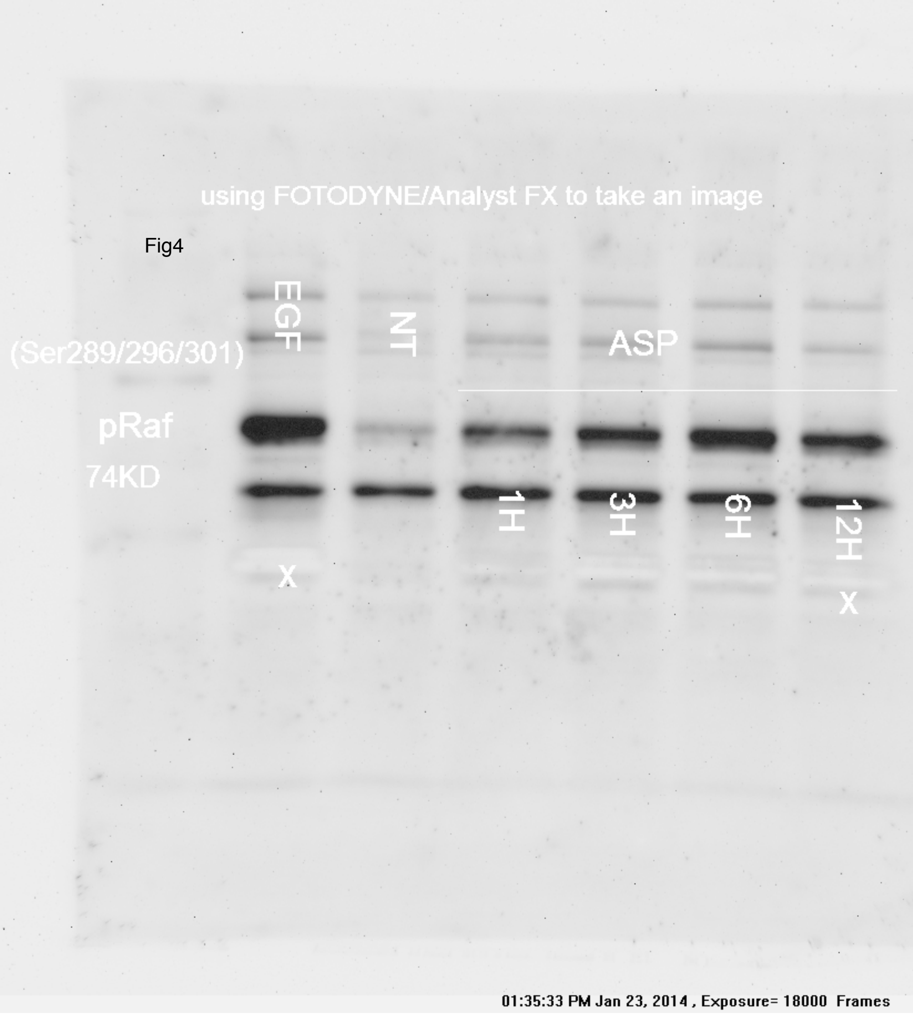

using FOTODYNE/Analyst FX to take an image
(Ser289/296/301)
pRaf
74KD
EGF
NT
ASP
1H
3H
6H
12H
X
X
01:35:33 PM Jan 23, 2014 , Exposure= 18000 Frames

Fig4

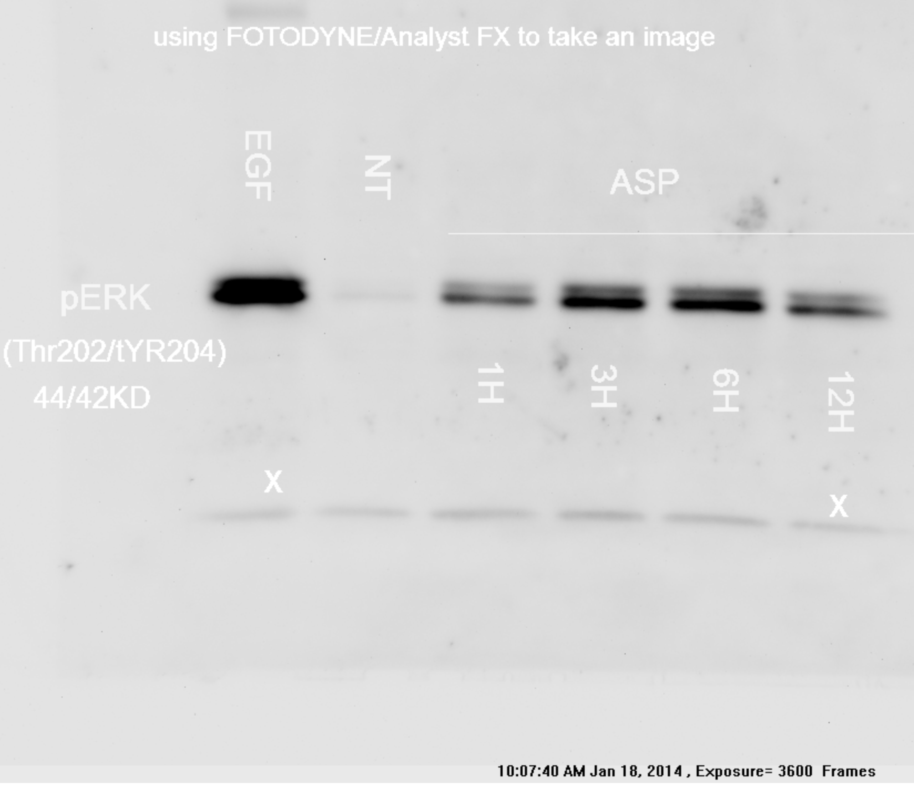
using FOTODYNE/Analyst FX to take an image
EGF
NT
ASP
pERK
(Thr202/tYR204)
44/42KD
1H
3H
6H
12H
X
X
10:07:40 AM Jan 18, 2014 , Exposure= 3600 Frames

using FOTODYNE/Analyst FX to take an image

Fig4

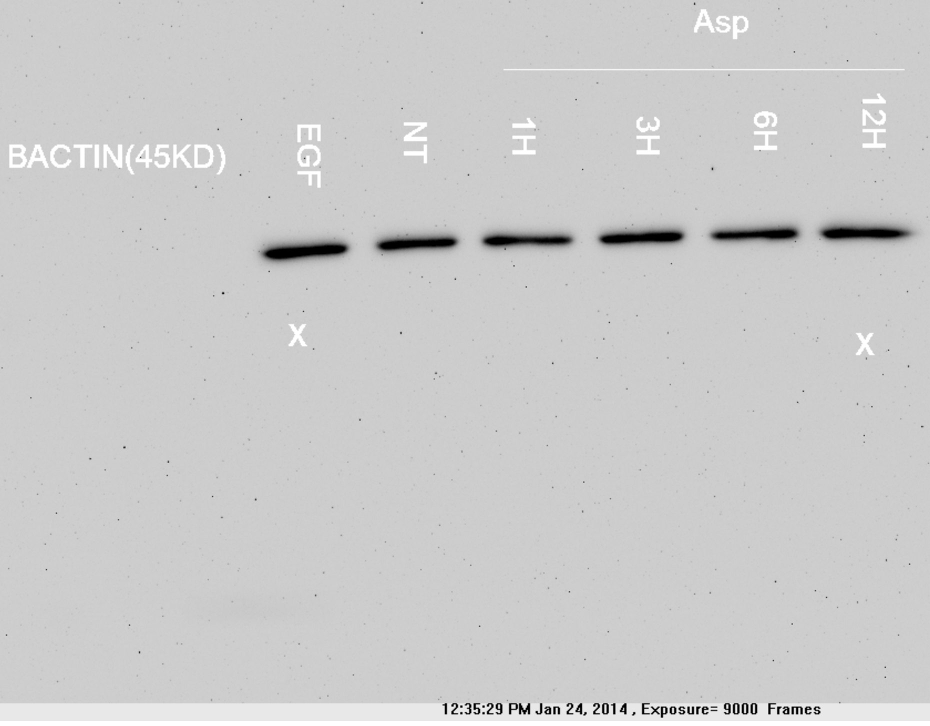

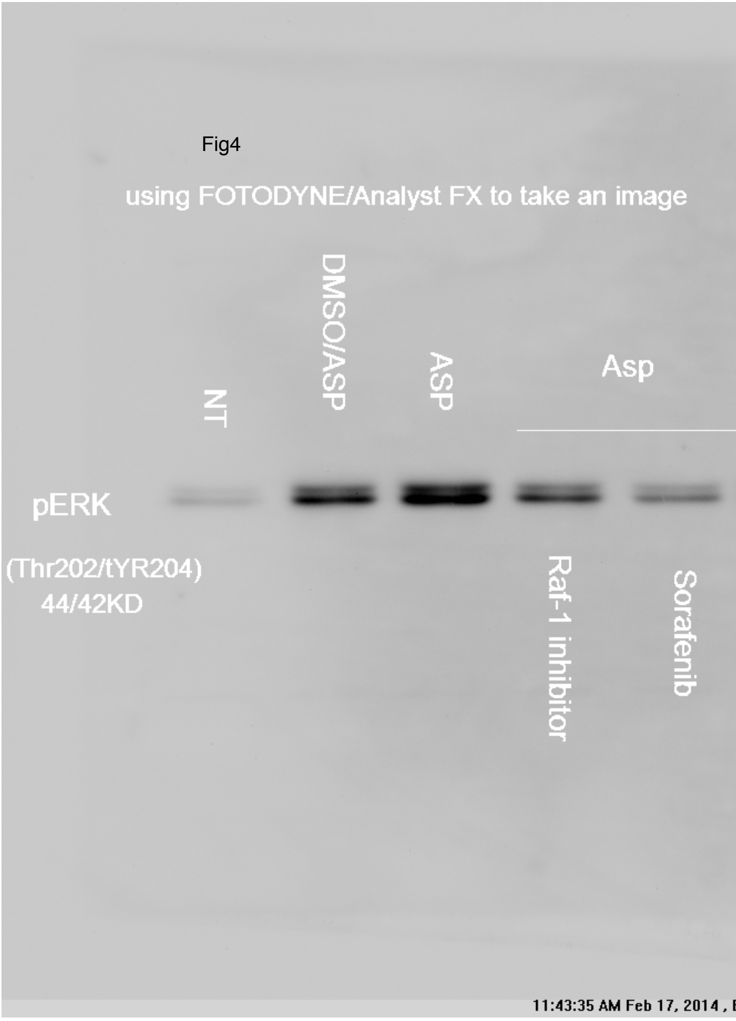
Fig4
using FOTODYNE/Analyst FX to take an image
NT
DMSO/ASP
ASP
Asp
pERK
(Thr202/tYR204)
44/42KD
Raf-1 inhibitor
Sorafenib
11:43:35 AM Feb 17, 2014 ,

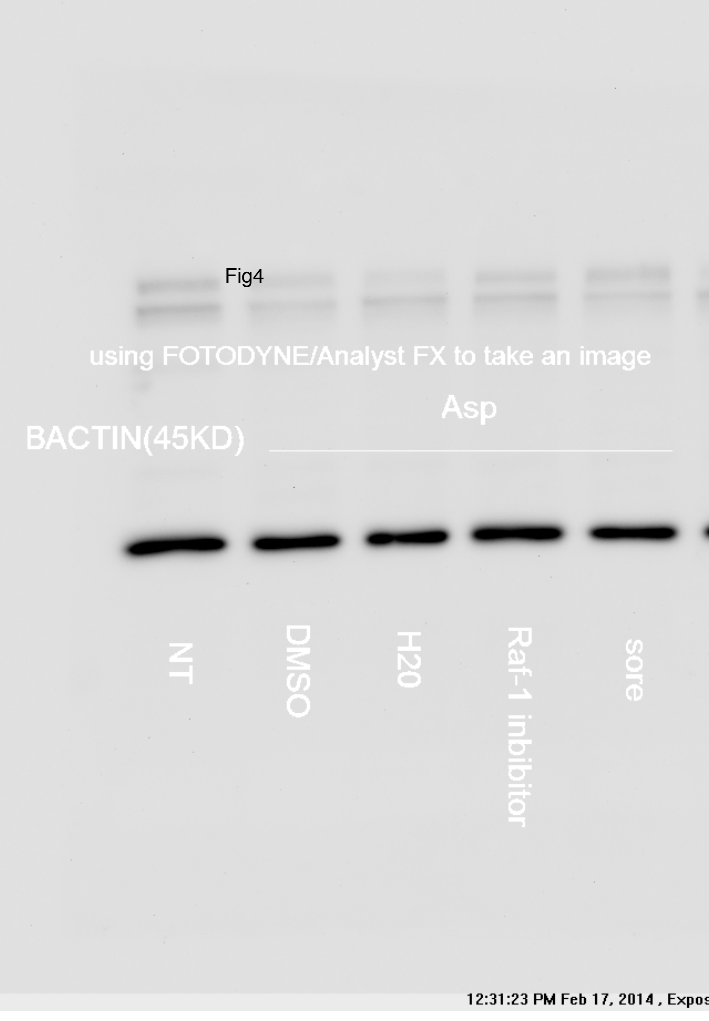
Fig4
using FOTODYNE/Analyst FX to take an image
Asp
BACTIN(45KD)
NT
DMSO
H20
Raf-1 inbibitor
sore
12:31:23 PM Feb 17, 2014 , Expos

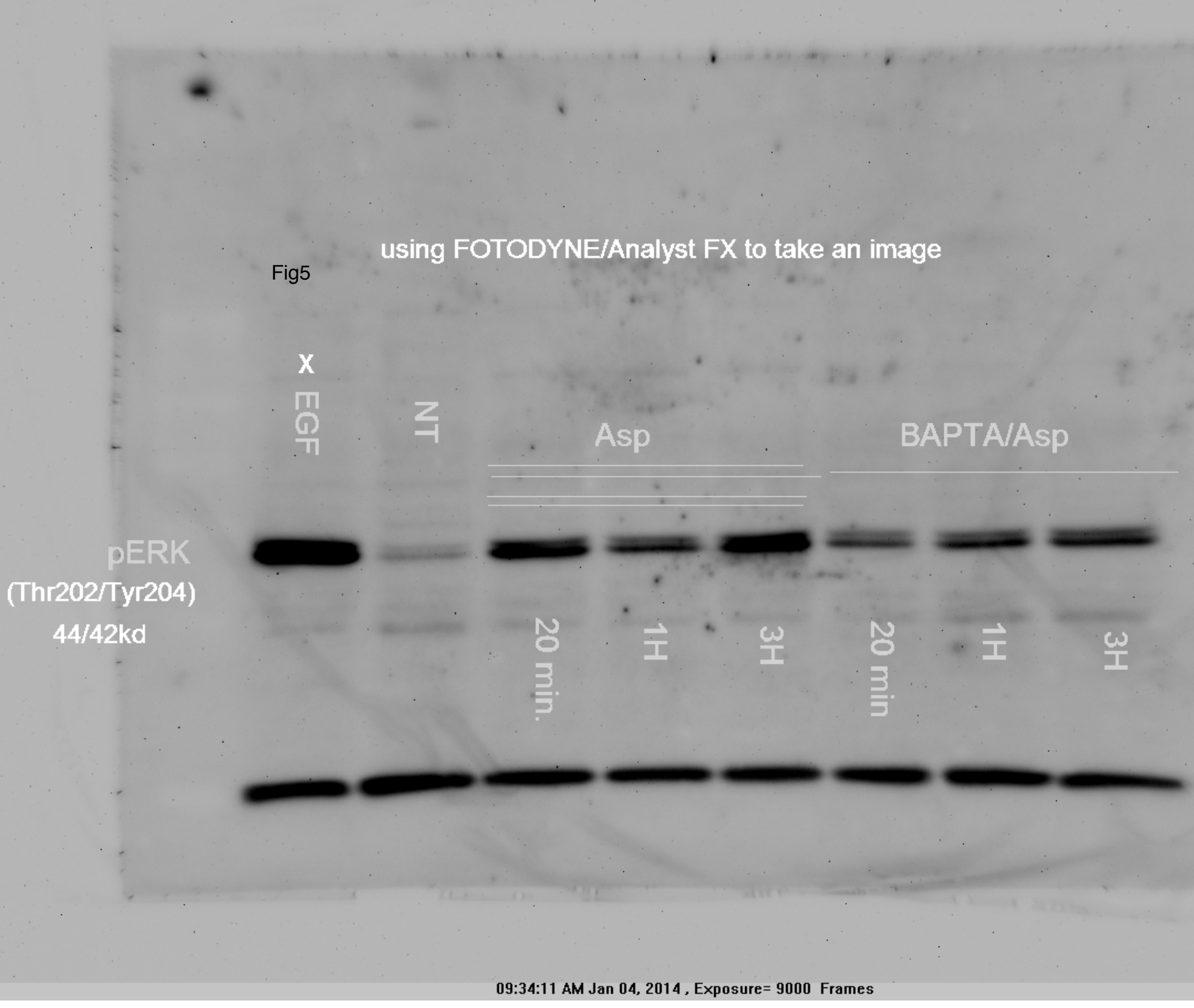

using FOTODYNE/Analyst FX to take an image
Fig5
X
EGF
NT
Asp
BAPTA/Asp
pERK
(Thr202/Tyr204)
44/42kd
20 min.
1H
3H
20 min
1H
3H
09:34:11 AM Jan 04, 2014 , Exposure= 9000 Frames

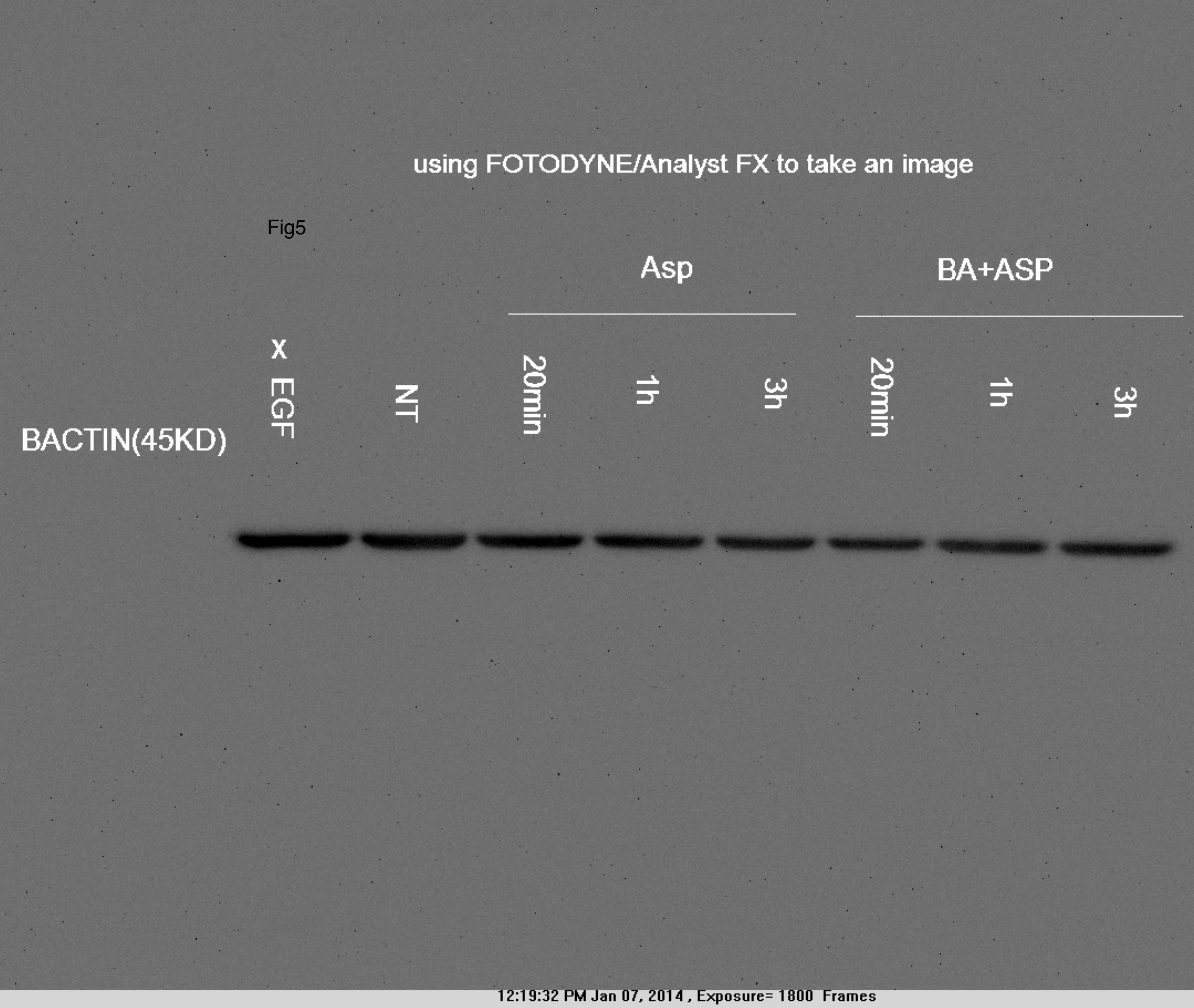
using FOTODYNE/Analyst FX to take an image
Fig5
Asp
BA+ASP
X EGF
NT
20min
1h
3h
20min
1h
3h
BACTIN(45KD)
12:19:32 PM Jan 07, 2014 , Exposure= 1800 Frames

Fig5

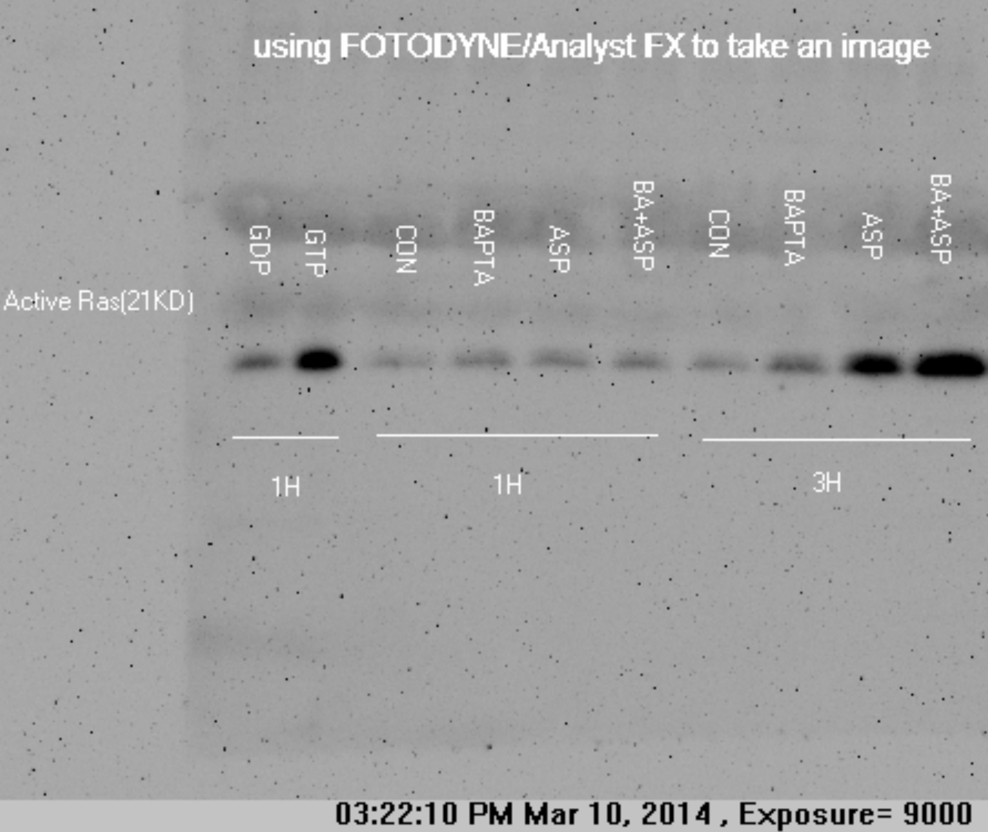
using FOTODYNE/Analyst FX to take an image
GDP
GTP
CON
BAPTA
ASP
BA+ASP
CON
BAPTA
ASP
BA+ASP
Active Ras(21KD)
1H
1H
3H
03:22:10 PM Mar 10, 2014 , Exposure= 9000
