# Supplementary material for: Exposure to mold proteases stimulates mucin production in airway epithelial cells through Ras/Raf1/ERK signal pathway
Source: PLoS One. 2020 Apr 22;15(4):e0231990. doi: 10.1371/journal.pone.0231990 (PMC7176129; doi:10.1371/journal.pone.0231990)
Supplement: Attachment — Submitted filename: Response to Reviewers.docx [file pone.0231990.s004.docx]

Dear reviewer:

Thank you for your advice, it was very helpful for me. Here are my answers. Reply=R

**Response for first review:**

1. Please ensure that your manuscript meets PLOS ONE's style requirements.

R: Yes, I have checked. My manuscript meets PLOS ONE's style requirements.

2. At this time, we ask that you please provide the product numbers and lot numbers of the A. fumigatus extracts purchased from GREER (Lenoir, NC), the inhibitors/neutralizzing antibodies and primary antibodies used in this study.

R: The product sheet is below.

3. Thank you for stating in your Funding Statement: "The study was supported partly by NIH grant AI39439.
The funders had no role in study design, data collection and analysis, decision to publish, or preparation of the manuscript."

Please also include the statement “There was no additional external funding received for this study.” in your updated Funding Statement.

Please include your amended Funding Statement within your cover letter. We will change the online submission form on your behalf.

R: Yes, I have corrected it in my cover letter and I have changed my Funding sourse.

4.About the WB original data. In your cover letter, please note whether your blot/gel image data are in Supporting. Information or posted at a public data repository.

R: I have provided my original blot data via Submission System and corrected it in my cover. letter.

5. PLOS requires an ORCID iD for the corresponding author in Editorial Manager on papers submitted after December 6th, 2016.

R: Here is my ORCID iD: https://orcid.org/0000-0002-2191-6873.

6. We note that you have included the phrase “data not shown” in your manuscript. Unfortunately, this does not meet our data sharing requirements. PLOS does not permit references to inaccessible data.

R: Yes, I have already corrected it.

7、Reviewer #1: The cells response to AFE in MUC5AC transcripts are more than 10 times higher in the 24 hours time-point. Please clarify why 6 hours time-point was chosen.

R: For the mechanistic study, the early event is usually driven by a few immediate signaling pathways that are easy to dissect. When the exposure time goes on, secondary or tertiary events triggered by initial signaling may complicate the overall data interpretation. Therefore, we tend to choose the time points as early as the responses are demonstrated.

8、Is the concentration of AFE physiologically relevant during fungal infections? Please clarify

why these doses were chosen.

R: We tested the dose response and found that this dose can induce high levels of mucin expression without outright cell death. In reality, the level of AFE exposure has been difficult to establish as its direct measurement is lacking. Nonetheless, AFE exposure model will lead to the discovery of mechanistic basis of Aspergillus induced illnesses.

9、It was mentioned earlier that the 6 hrs time-point was selected. Please clarify why showing

the 24 hrs time-point in figure 1? Please quantify MUC5AC and MUC5B protein levels for. figure 1.

R: The 24hr time point is shown to demonstrate that this effect is time-dependent. The quantified protein levels are shown in Fig 1F.

10、What are the diluents of EGFR inhibitors?

R: DMSO.

11、Do cells survive for 24 hrs after the EGFR inhibitor treatment? An LDH cytotoxicity assay

will determine how toxic these inhibitors are to the cells.

R: Yes, cells still survive. Related data is in S1_File.

12、At which time-point cells were stained in figure 5 E?

R: I think you mean Fig 5A, cells were pre-treated with 50 uM BAPTA-AM for 30 min and. then used 5ul Fluo-4/AM in 1ml OPTI-MEM for another 30 min. After, wash 2x with OPTI-MEM (DMEM/HEPES/no phenol) and let it sit in fresh DMEM for another 30 minutes to allow the ester to cleave. At last, they were treated AFE for 3, 6,9, 10 min.

14、LDH cytotoxicity assay should be performed for figure 3 to determine the toxicity of

incubating cells with trypsin for 6 hrs.

R: Related data is in S1_File.

15、Graph labels are not clear figure 3 B-C.

R: Thank you, I have corrected this.

16、What percentage of DMSO was used in figure 4? Is this amount of DMSO toxic to cells?

LDH cytotoxicity assay will answer this question.

R: The maximum percentage is 0.3% for U0126 inhibitor in this experiment. Related data is in S1_File.

17、Please clarify why MUC5B data is not shown in figure 5?

R: Because 5B induction by AFE treatment is a little bit low, we didn’t show it at first, but I. have added this data in Fig 5C.

18、Please highlight the significance of this work and how it contributes to the asthma research

field in the discussion section.

R: Please see the highlight part in revised manusription.

**Response to second review:**

1) Please provide additional information about each of the cell lines used in this work, including any quality control testing procedures (authentication, characterisation, and mycoplasma testing). For more information, please see <http://journals.plos.org/plosone/s/submission-guidelines#loc-cell-lines>.

R: I have add relevant information in Cell line section.

2) To comply with PLOS ONE submission guidelines, in your Methods section, please provide additional. information regarding your statistical analyses. For more information on PLOS ONE's expectations for statistical reporting, please see <https://journals.plos.org/plosone/s/submission-guidelines.#loc-statistical-reporting>.

R: I have corrected my statistical analyses in your Methods section.

3) At this time, we ask that you please add scale bars to the microscopy images presented in Figures 1. and 5 and refer to the scale bar in the corresponding Figure legend.

R: Yes, I have add the scale bar analyses in Figures 1. and 5.

Product name	Product number	Lot number

Aspergillus fumigatus	XPM3C3A25	231795

AG1478	658548-1MG	D00108234

U0126	662005-1MG	D00122919

BIBX 1382	324832-5MG	B77319

neutralizing anti-EGFR Ab	05-101	0120201A1

Raf-1 inhibitor	553003

Sorafenib	S-8502	BSF-106

PMSF	P7626-5G	BCCB9214

Glutathione reduced ethyl ester	G1404-25MG	BCCB5246

PORCINE Trypsin	85450C-25MG	12N438AR1C

EGF	01-407	DAM1687496

BAPTA/AM	196419-25MG	3278031

pERK1/2 (T202/Y204) Ab	9101S	15

pRaf-1 (Ser289/296/301) Ab	9431S	2

anti-PAR2 neutralizing (SAM11) Ab	SC-13504	C0713

anti-MUC5B (H-300) Ab	SC -20119	F0611

anti-pEGFR (Y1173) Ab	SC -12351	E2813

anti-EGFR (1005) Ab	SC -03	H1007

anti-pTyr (py99) Ab	SC -7020	L1106

β- ACTIN (C4) Ab	SC -47778	H0515

anti-MUC5AC Ab	MS-145-PI	145P1405I

EnzChek® Protease Assay Kit	E6638	1445257

RAS GTPASE PULLDOWN Kit	PI16117	PA199259

Promega* Dual-Glo* Luciferase Assay	PR-E2940	0000303692

Cell Proliferation Assay Kit	G3580	0000328878

Fluo-4/AM	F14201	2165131
